# Proteomic and metabolomic signatures of U87 glioblastoma cells treated with cisplatin and/or paclitaxel

**DOI:** 10.1080/07853890.2024.2305308

**Published:** 2024-01-22

**Authors:** Munazza Ahmed, Ahlam M. Semreen, Alexander D. Giddey, Wafaa S. Ramadan, Raafat El-Awady, Nelson C. Soares, Waseem El-Huneidi, Yasser Bustanji, Mohammad A. Y. Alqudah, Karem H. Alzoubi, Mohammad H. Semreen

**Affiliations:** a Department of Pharmacy Practice and Pharmacotherapeutics, College of Pharmacy, University of Sharjah, Sharjah, United Arab Emirates; b Research Institute of Medical and Health Sciences, University of Sharjah, Sharjah, United Arab Emirates; c Department of Medicinal Chemistry, College of Pharmacy, University of Sharjah, Sharjah, United Arab Emirates; d Laboratory of Proteomics, Department of Human Genetics, National Institute of Health Doutor Ricardo Jorge (INSA), Lisbon, Portugal; e Department of Basic Medical Sciences, College of Medicine, University of Sharjah, Sharjah, United Arab Emirates; f Department of Basic and Clinical Pharmacology, College of Medicine, University of Sharjah, Sharjah, United Arab Emirates; g School of Pharmacy, The University of Jordan, Amman, Jordan; h Department of Clinical Pharmacy, Faculty of Pharmacy, Jordan University of Science and Technology, Irbid, Jordan

**Keywords:** Cisplatin, glioblastoma, GBM, paclitaxel, proteomics, systems biology, uhplc-qtof-ms, untargeted metabolomics

## Abstract

**Background:**

Glioblastoma (GBM) is a primary malignancy of the central nervous system and is classified as a grade IV astrocytoma by the World Health Organization (WHO). Although GBM rarely metastasizes, its prognosis remains poor. Moreover, the standard treatment for GBM, temozolomide (TMZ), is associated with chemoresistance, which is a major factor behind GBM-related deaths. Investigating drugs with repurposing potential in the context of GBM is worthwhile to bypass lengthy bench-to-bedside research. The field of omics has garnered significant interest in scientific research because of its potential to delineate the intricate regulatory network underlying tumor development. In particular, proteomic and metabolomic analyses are powerful approaches for the investigation of metabolic enzymes and intermediate metabolites since they represent the functional end of the cancer phenotype.

**Methods:**

We chose two of the most widely prescribed anticancer drugs, cisplatin and paclitaxel. To our knowledge, the current literature lacks studies examining their effects on metabolic and proteomic alterations in GBM. We employed the mass spectrometry technological platform ‘UHPLC-Q-TOF-MS/MS’ to examine the changes in the proteome and metabolome profiles of the U87 cell line with defined concentrations of cisplatin and/or paclitaxel *via* an untargeted approach.

**Results:**

A total of 1,419 distinct proteins and 90 metabolites were generated, and subsequent analysis was performed. We observed that upon treatment with cisplatin (9.5 μM), U87 cells exhibited apparent efforts to cope with this exogenous stressor, understanding the effect of paclitaxel (5.3 μM) on altering the transport machinery of the cell, and how the combination of cisplatin and/or paclitaxel suggests potential interactions with promising benefits in GBM therapeutics.

**Conclusion:**

Our research provides a detailed map of alterations in response to cisplatin and paclitaxel treatment, provides crucial insights into the molecular basis of their action, and paves the way for further research to identify molecular targets for this elusive malignancy.

## Introduction

Glioblastoma (GBM) is an aggressive primary brain malignancy classified by the WHO as a grade IV astrocytoma [1]. With a global incidence of below 0.01%, GBM is more commonly known for its poor prognosis [2]. In fact, for most GBM patients, the prognosis after diagnosis is at most one year [3]. Currently, there are no therapies for GBM, and the standard of care for most patients with newly diagnosed GBM is surgery, followed by radiation and chemotherapy with temozolomide (TMZ), an alkylating agent [4, 5]. Although the standard of care adds to the survival advantage, surgical resection is usually associated with neurological complications [6] and TMZ with resistance; over 50% of GBM patients eventually fail to respond to therapy [7].

Although significant efforts have been made to investigate novel therapeutic strategies in fields such as immunotherapy and precision oncology, biological characteristics, including the blood-brain barrier (BBB) and distinct tumor and immunological milieu, provide substantial obstacles to the development of innovative treatments. In addition, the road to discovering and bringing novel therapeutics from bench to bedside can become exhaustive. Therefore, it is worthwhile to explore other therapeutic options for GBM that have the potential to be repurposed. Our research is centered on cisplatin and paclitaxel; two established salvage anti-cancer medications that have been evaluated in clinical trials for glioma patients [8, 9].

### Cisplatin

Cisplatin belongs to the same class as TMZ; however, it is a platinum-based chemotherapeutic drug. Testicular cancer, bladder carcinoma, and advanced ovarian cancer are among the conditions for which cisplatin is FDA-approved [10, 11] .Yet when the advantages outweigh the dangers of adverse effects, oncologists use cisplatin outside these guidelines to treat malignancies such as recurrent brain tumors [12]. Nonetheless, its clinical use is limited because of the adverse side effects of long-term treatment regimens associated with GBM [13]. Furthermore, while platinum-based compounds can cause side effects such as nephrotoxicity and neurotoxicity, their safety and efficacy profile is well established [14], and toxicity is manageable with appropriate dosing and monitoring [15]. In this context, clinical trials in gliomas report the treatment to be well tolerated, with no systemic or local harm noted [16, 17]. In one of the studies, for instance, patients implanted with biodegradable polymers and cisplatin upon the removal of primary GBMs exhibited an overall survival of 14.2 months as opposed to 7 months in control subjects [17].

### Paclitaxel

Paclitaxel is a microtubule inhibitor that stabilizes polymerized microtubules during mitosis, eventually leading to cell death [18]. It is clinically used in the treatment of ovarian, breast, pancreatic and lung cancers [19–22]. Although paclitaxel possesses one of the strongest anti-glioma actions *in vitro* [23], it is unable to penetrate the BBB and reach infiltrative glioma cells at significant concentrations. This, however, can be mitigated through the utilization of pharmaceutical delivery systems such as liposomes and nanosomes [24]. Paradoxically, paclitaxel treated cells have been shown to escape mitotic arrest and undergo abnormal mitosis due to multi-polar division of the resultant multi-nucleated cells [25]. Therefore, it can be suspected that paclitaxel could be effective for cancer that exhibits a deformed nuclear envelope. Recently, a multi-omics-based investigation identified a previously unidentified role for lamin genes (which encode for the primary elements of the nuclear matrix) in gliomagenesis, giving compelling evidence for the crucial connection between the survival of glioma patients and abnormal tumor nuclear structure [26]. These findings challenge the traditional understanding of paclitaxel’s mechanism of action and suggest that it may have other targets within the cell.

### The combination

Although the majority of cancer patients respond well to platinum, many will eventually develop cisplatin-resistance, precipitating tumor relapse [27]. Nonetheless, earlier studies have reported paclitaxel to be effective in treating gliomas that relapse following alkylating agent-based chemotherapy [28, 29]. While the combination of cisplatin and paclitaxel for GB patients is unexplored in the literature, paclitaxel could improve the therapeutic efficacy of alkylating drugs while avoiding cross-resistance [23]. A multicenter trial demonstrated that cisplatin and docetaxel, exhibit significant efficacy in treating advanced gastric cancer, with a high response rate and minimal adverse effects [30]. Moreover, another study reported that a combination of cisplatin and paclitaxel had lower toxicity profiles in patients with ovarian cancer [31]. While there is scarce literature on the effect of both cisplatin and paclitaxel in the context of GBM, their combination therapy has been shown to have a lower toxicity profile in patients with ovarian cancer [31], which brings into question the therapeutic utility of this combinatorial effect on GBM. In this regard, there is currently a lack of literature regarding their molecular changes in GB and the identification of molecular changes in response to the selected treatment(s) could offer profound insights into the therapeutic targets of drugs, as well as aid in evaluating patient therapy to improve clinical prognosis.

## Materials and methods

### Reagents

Fetal bovine serum (FBS), penicillin, streptomycin, trypsin, tryptan blue, Bradford’s reagent, phosphate-buffered saline (PBS), cisplatin, and paclitaxel were acquired from Sigma-Aldrich (Darmstadt, Germany). Dulbecco’s modified Eagle’s medium (DMEM) was purchased from PAN Biotech (Aidenbach, Germany). Acetonitrile (ACN), deionized water, LC-MS CHROMASOLV, and methanol (≥99.9%) were purchased from Honeywell (Charlotte, USA). Formic acid (FA) and trifluoroacetic acid (TFA) were procured from Fisher Scientific (Loughborough, UK), whereas hydrochloric acid (HCl, 37%) was sourced from VWR Chemicals (Paris, France). C18 STAGE (stop and go extraction) tips, lysis buffer, lysyl-endopeptidase (Lys-C), and Pierce^TM^ protease inhibitor tablets were purchased from Thermo Fisher Scientific (Rockford, USA).

### Cell line and culture conditions

This study employed the Uppsala 87 Malignant Glioma (U87) cell line. The decision to use a single-cell line is driven by the advantages it offers, including genetic homogeneity, streamlined experimental design, improved resource utilization, and comprehensive molecular characterization. Despite several large-scale chromosomal abnormalities, U87 has exhibited a rather stable chromosomal structure over time, implying that previous research on U87 cells can be reevaluated [32]. Additionally, other GBM cell lines, such as U251 and U373, exhibit higher intrinsic resistance to TMZ [33], making U87 a suitable option to prevent confounding results that could occur from the use of cisplatin. This cell line is well established in the literature and was purchased from the American Type Culture Collection (ATCC) (Manassas, USA) (RRID:CVCL_0022). In the DMEM culture containing 10% fetal bovine serum and 1% penicillin/streptomycin, the U87 cell line was cultivated as monolayers. Each cell culture was maintained at 37 °C in a humid environment with 5% CO_2_, with the culture media being replaced every two to three days. The number of viable cells was estimated using a hemocytometer.

### Treatment of cells with anticancer drugs

Two million cells were seeded into T-75 cell culture flasks and incubated overnight. Control cells were treated with 0.5% Dimethyl sulfoxide (DMSO) for 24 h. U87 cells were treated with cisplatin at a concentration of 9.5 μM and/or paclitaxel (5.3 μM for a period of 24 h. These concentrations were obtained from the literature based on the IC_50_ values of the drugs investigated in the U87 cell line [9, 34]. After incubation, the cells were harvested by trypsinization, washed twice with PBS, and resuspended in 1 mL of PBS for analysis. Subsequently, the cells were centrifuged at 1200 rpm for 10 min at room temperature and the resultant pellets were collected. The resultant four groups with two biological replicates were stored at −80 °C for subsequent metabolomic and proteomic analyses.

### Metabolomics and proteomics analysis

The procedures involved in sample preparation before LC-MS/MS analysis are described below. The protocol followed is an established procedure because of its ability to have a higher coverage of extracted metabolites [35].

#### Sample extraction for proteomics and metabolomics

Frozen cell pellets from the respective treatments were thawed and spun at 4600 rpm for 5 min at 4 °C to separate the cells from the PBS buffer, which was then discarded. The pellets were subjected to 400 μL of lysis buffer, prepared by dissolving one protease inhibitor tablet in 10 mL of lysis buffer for 10 min. The samples were then ultrasonicated to destroy insoluble matrices. The remaining cell debris was removed after the samples were centrifuged at 14000 rpm for 5 min at 4 °C, whereby the supernatants were transferred to Eppendorf tubes (a). Next, 400 μL of methanol, followed by 300 μL of chloroform, were added to (a) and vortexed to mix the resultant solution. They were then centrifuged at 14000 rpm for 5 min, which yielded two metabolite-containing layers separated by a protein disk. To circumvent the disk and obtain the metabolite-rich layer, the upper layer was pipetted and placed in LC glass vials, whereas the residual white disk in (a) was subjected to 300 μL of methanol, vortexed, and centrifuged at 14000 rpm for 5 min to precipitate the protein. The supernatant was transferred to the metabolite-containing glass vials to evaporate the solvents using EZ-2 Plus evaporator at an estimate of 34 °C and 13 mbar (GeneVac, Ipswich, UK) for 2 h, and the protein pellets were air dried for an estimate of 3 h.

### Proteomics

#### Proteomics sample preparation

The protein samples were air-dried and subsequently reconstituted in 100 μL denaturation buffer consisting of 6 M urea and 2 M thiourea in 10 mM Tris(hydroxymethyl)aminomethane (Tris) buffer (pH 8). Protein quantification and digestion were performed using a modified Bradford assay [36] and in-solution digestion, respectively.

#### In-solution protein digestion and peptide desalting

Dithiothreitol (DTT) was used to reduce protein samples (100 μg) and break disulfide bonds, which were then incubated for an hour at room temperature (RT) with agitation at 100 rpm. The samples were then alkylated with iodoacetamide (IAA) (5.5. mM), and incubated for 1 h at 100 rpm in the dark. Following the addition of 1 μg LysC (1:100 w/w), the samples were incubated for 3 h at 100 rpm at room temperature. The samples were then diluted four times with ammonium bicarbonate (20 mM), digested with trypsin (1 ug) (1:100 ratio), and incubated overnight at RT and 100 rpm. Next, the samples were dried, resuspended in 1% trifluoroacetic acid (TFA), and filtered using C18 StageTips for desalting. By eliminating contaminants such as gel fragments and aggregates, which could block the column, cleaning the peptides on a StageTip ensures the stability of the LC-MS/MS system [37].

#### LC-MS/MS conditions

Our samples were analyzed using the UHPLC elute paired with a hybrid of Q-TOF-MS (UHPLC-Q-TOF/MS). The instruments and components were obtained from Bruker Daltonics (Bremen, Germany). Following sample resuspension, 10 μL of sample was added to the LC-MS/MS system. Then, reverse-phase liquid chromatographic separation was carried out using an Intensity Solo HPLC column (100 × 2.1 mm, 1.8 m). FA (0.1%) in HPLC-grade water (referred to as ‘A’) and 0.1% FA in ACN (‘B’) comprised the mobile phase. For peptide separation, gradient elution was used as follows, with a constant flow rate throughout of 300 nL/min *and column temperature of 35 °C*: 5% B for 5 min, 35% B over 85 min, then 95% B over 5 min, maintained for 5 min, then 10 min of column re-equilibration. During separation, the back pressure values were < 350 bar.

#### Bioinformatics analysis and statistical approach

To identify the proteins and peptides, the raw data were processed using MaxQuant (version 1.6.17.0) software (https://www.maxquant.org/) using the UniProt proteome for Homo sapiens (Proteome ID: UP000005640, 81,791 entries, 23 March 2023) and the Andromeda search engine. In the MS/MS database search, default parameters were used, including variable modifications, such as methionine oxidation and acetylation of protein N-termini, and fixed modifications, such as carbamidomethylation of cysteine residues. Peptide spectral matching (PSMs) was performed using a 20-ppm precursor mass tolerance and a 1% false discovery rate (FDR). The MaxLFQ algorithm was used for label-free quantification (LFQ). The default trypsin/P enzymatic cleavage rule was used for in silico digestion. The data were filtered to remove potential contaminant proteins and proteins solely recognized by the site and reverse proteins.

The LFQ data were transformed into log_2_ (x). Proteins were annotated and those with 70% valid values were used for further analysis. To determine the substantially expressed proteins in treated and untreated U87 cells, a two-tailed independent Student’s t-test was used. The Benjamini-Hochberg method was used for multiple testing corrections. If the adjusted *p*-value was < 0.05, and the log_2_ fold change was > 1, proteins were considered to be differentially expressed. To evaluate the expression of differentially expressed proteins in each group and to show group clustering, hierarchical clustering and a heat map were generated. More importantly, dysregulated proteins with an adjusted *p*-value < 0.05 were examined for enrichment by the Gene Set Enrichment Analysis (GSEA) through R software programming to display the gene ontology (GO) keywords for biological processes.

### Metabolomics

#### LC-MS/MS conditions

After resuspension of the dried samples with 200 μL of 0.1% FA in water, 10 μL was injected for LC-Q-TOF/MS sample analysis. Although the mobile phase used was identical to that used for proteomics, the flow rate was fixed at 0.25 ml/min for elution and altered for recalibration. The gradient elution was carried out as follows: after holding 1% B for 2 min, 99% B over the course of 15 min, and then plateaued at 99% B for 3 min, 1% B was re-equilibrated for 10 min. The Electrospray ionization (ESI) capillary voltage was 4500 V and its drying temperature was 220 °C. A QTOF mass spectrometer was used to obtain mass spectra in a data-dependent manner, automatically switching between MS and MS/MS scans between 20 and 1300 m/z.

#### Bioinformatics analysis and statistical approach

MetaboScape® 4.0 program was used for metabolomic analysis (Bruker Daltonics, Bremen, Germany). A minimum peak length of seven spectra and a minimum intensity threshold of 1000 counts for peak identification were used as parameters to identify molecular features. To recalibrate the mass spectra, the injected external calibrant was utilized in the range of 0–0.3 min, and the peak area was used for quantification. The chosen mass-to-charge ratio (m/z) ranged from 50 to 1000, while the retention time for scanning was 0.3 to 25 min. Only features that appeared in at least 3 of the 16 samples were considered for further evaluation. Using retention time and matching with MS^2^ spectra, MetaboScape® identified the metabolites by comparing them to the human metabolome database (HMDB). When more than one characteristic matched a particular database entry, the annotation quality (AQ) score was applied to select the best match. By considering which features best fit the combined MS/MS, precursor m/z values, retention duration, and isotope pattern scores, a higher AQ score denoted the best overall match.

software MetaboAnalyst 5.0 (https://www.metaboanalyst.ca), a comprehensive platform for metabolomic data analysis, was used to import the metabolite datasets once they were exported as a CSV file. The rationale behind the selection of this software stems from its ability to offer a mostly automated approach as opposed to its competitors. Additionally, the outcomes of spectrum processing can be quickly transmitted to additional MetaboAnalyst-compatible modules for statistical and functional analysis [38]. The metabolomic data underwent data transformation (log transformation of base 10) to lessen the influence of outliers and enhance the normality of the data distribution. The false discovery rate (FDR) approach was used to correct multiple hypothesis testing and to lower the number of false positives. Substantially altered metabolites in the treatment groups were determined using a two-tailed independent Student’s t-test in comparison to the control group. Consequently, a heatmap with hierarchical clustering (*p*-value < 0.05) and volcano plots (*p*-value< 0.05, fold change threshold of 1.5) were developed to demonstrate significant alterations in cellular metabolites. Functional enrichment was generated for the significant metabolite sets (*p* < 0.05).

### Joint pathway analysis

Joint pathway analysis was opted for multi-omics integrated analysis, given its ability to integrate diverse molecular data types and biologically relevant information through the identification of pathways and networks that are dysregulated across multiple omics layers. By leveraging the complementary strengths of proteomics and metabolomics, joint analysis can enable a holistic understanding of the effects of our intervention on the GBM cells. This analysis was conducted using MetaboAnalyst (https://www.metaboanalyst.ca/MetaboAnalyst/). The corresponding analysis tool was employed to show the enriched pathways for each treatment condition using the option ‘all-pathways,’ which considers the regulatory and metabolic pathways. The Uniprot IDs of significant proteins (*p* < 0.05) and compound names of the significant metabolites (*p* < 0.05) for each treatment group were submitted for analysis.

## Results

### Proteomics analysis

#### Data quality assessment

**Figure 1.**
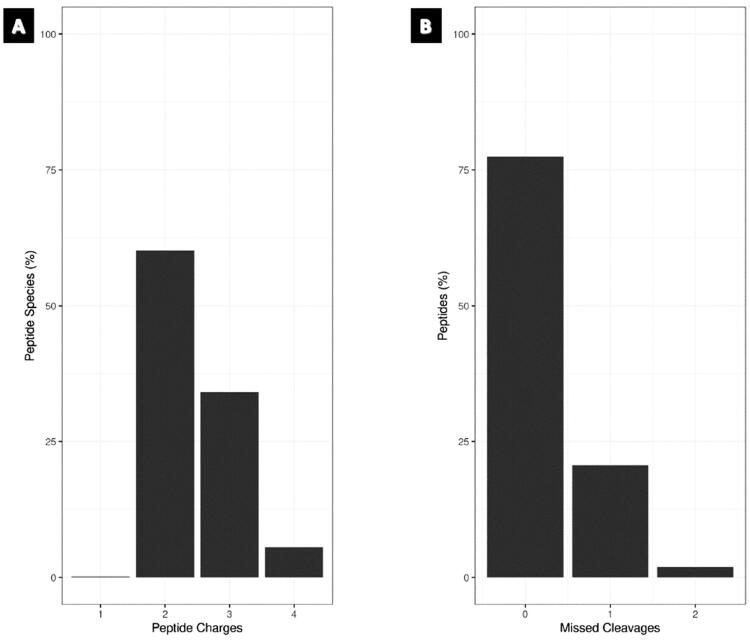
Bar graph – QC metrics for the proteomics data. (A) Charge state distribution of identified peptides across all samples. (B) QC miscleavage analysis, showing the percentage of peptides with zero, one and two missed cleavages.

Quality control (QC) cleavage and QC charge were assessed to evaluate the reliability of proteomics data. More than 75% of the peptides were fully cleaved, indicating efficient digestion and high confidence in peptide identification, while more than 60% of the detected peptides had a charge state of 2^+^, according to QC charge analysis, with the majority of peptides having the correct charge assignment (Figure 1). These findings offer assurance in the peptide identification and quantification of our sample and demonstrate the dependability and correctness of our proteomics data.

#### Raw LC-MS/MS results

Herein, LC-MS/MS was used to perform a discovery proteomics study along with an untargeted metabolomics analysis on U87 cells treated with cisplatin and/or paclitaxel versus cells treated with DMSO to understand the molecular changes of cisplatin and/or paclitaxel on GBM cells. Match between run analysis was conducted to align and compare protein abundance data between the two biological replicates, with one repeated injection collapsed to minimize technical variability. In our comparative proteomic analysis, 152,598 spectra were identified, resulting in 16,328 non-redundant peptides, 15,351 of which were specific to protein groups. A total of 2,208 proteins were identified from these peptides with an FDR of 0.95% for protein identifications. Moreover, a total of 1,419 distinct protein group assignments were retained after filtering out all protein groups other than those identified by at least one unique and two total peptides to provide reliable LFQ. Significance was determined by one-way analysis of variance (ANOVA) with a *p*-value of < 0.05, which resulted in 125 significantly altered proteins.

#### Differential abundance analysis

##### Heatmap analysis

**Figure 2.**
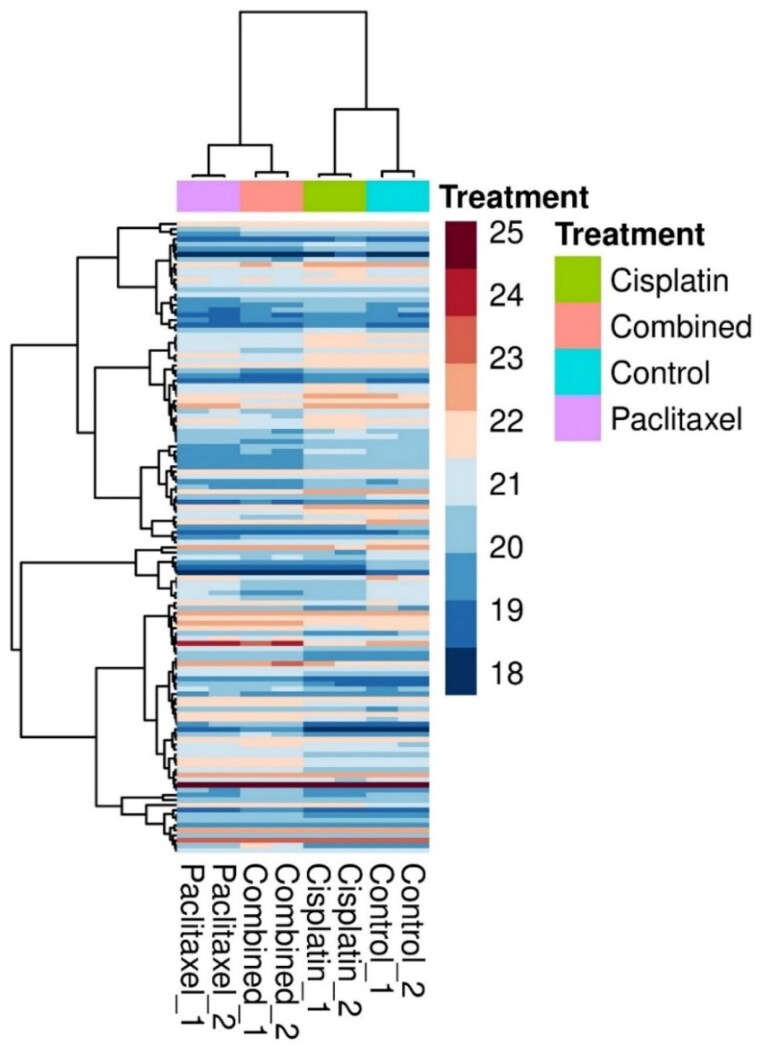
A hierarchical clustering heatmap of the deregulated proteins. Each column represents U87 upon treatment with cisplatin (9.5 μM), paclitaxel (5.3 μM) and combined (cisplatin (9.5 μM) and paclitaxel (5.3 μM)), while the rows represent the proteins. The color scale represents the log_2_-transformed abundance of the proteins from dark blue (down-regulated) to maroon (up-regulated).

To compare the expression levels of proteins across the treatment groups and control and identify clusters that were upregulated or downregulated in response to treatment, a hierarchical clustering heatmap of the 125 proteins was generated (Figure 2), which showed that paclitaxel and the combination treatment groups had a similar expression profile as that of the cisplatin-treated groups.

#### Analysis of variance

**Figure 3.**
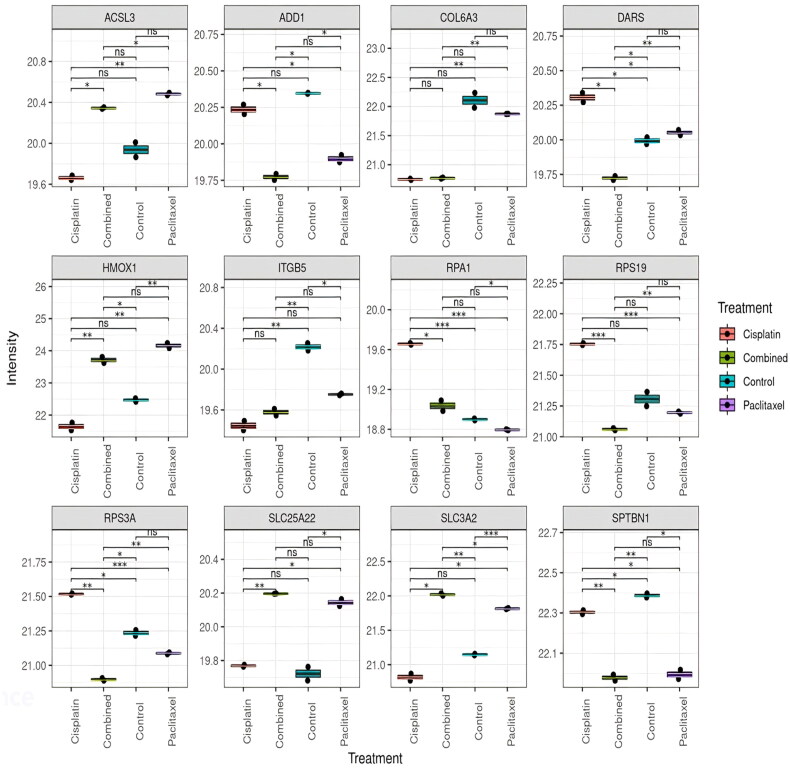
ANOVA box plot results representing the top 12 deregulated proteins across all samples. ANOVA was used to determine which proteins are significantly differentially expressed across all four groups, and can account for variability within and between groups, allowing for a comprehensive analysis of the differences between groups. X axis represents the different groups, and y axis represents log2 transformed LFQ intensity (*p < 0.05; **p < 0.01).

Extracting from the list of significantly altered proteins, Figure 3 represents the top 12 proteins dysregulated by the treatments from ANOVA. Table 1 lists the protein characteristics.

**Table 1.** Summary of proteins deregulated upon treatment with cisplatin and/or paclitaxel.

Gene	Protein Uniprot ID	Protein Name	GO -Biological Process * (Source – Uniprot)	Association with glioblastoma and/or other cancer(s)
ACSL3	A0A7P0TA76	Acyl-CoA Synthetase Long Chain Family Member 3	Cellular membrane component involved in lipid metabolic process.	According to the Cancer Genome Atlas, ACSL3 expression is reported to be 8% lower in GBM than in non-tumor cells. Poorer clinical outcomes have been linked to ACSL3 overexpression in patients with high-grade NSCLC [39].
ADD1	E7EV99	Alpha-adducin	Membrane-cytoskeleton-associated protein that supports the formation of the spectrin-actin network.	In basal cell carcinoma and squamous cell carcinoma, ADD1 is increased, implying that ADD1 participates in cell proliferation [40].
COL6A3	P12111	Collagen alpha 3(VI) chain	A cell-binding protein involved in cellular adhesion.	Highly expressed across multiple malignancies and has a significant impact on the progression of cancer [41].
DARS	P14868	Aspartate--tRNA ligase, cytoplasmic	Involved in catalyzing specific attachment of an amino acid to its corresponding tRNA.	DARS has been identified as a prognostic biomarker for GBM; its expression is correlated to tumor purity [42].
HMOX1	P09601	Heme oxygenase 1	Provides defense against programmed cell death, and this cytoprotective action is dependent on its capacity to catabolize free Heme.	In GBM, TGF-responsive HMOX1 expression is linked to stemness and invasion [43].
ITGBM5	P18084	Integrin beta 5	Receptor for fibronectin involved in cell-adhesion.	ITGBM5 is a possible target for the treatment of GBM and may serve as a predictive biomarker for GBM patient survival [44].
RPA1	P27694	Replication protein A1	Involved in cellular response to DNA damage, specifically involved in base-excision repair.	RPA is expressed by GBM stem cells (GSCs), and high RPA expression correlates with poor glioma patient survival [45].
RPS19	P39019	40S ribosomal protein S19	Required for the development of 40S ribosomal subunits and pre-rRNA processing and is also involved in extra-cellular functions of the immune system.	RPS19 is implicated in the pathophysiology of GBM after co-expression network analysis with a focus on 791 immune system-related genes [46].
RPS3A	P61247	40S ribosomal protein S3a	Structural constituent of ribosome involved in negative regulation of apoptotic process and translation initiation.	Over expression of RPS3A could be associated with the development of GBM [47].
SLC25A22	A0A0D9SFE1	Mitochondrial glutamate carrier 1	Involved in transmembrane transport.	SLC25A22 is upregulated in GBM [48].
SLC3A2	P08195	4F2 cell-surface antigen heavy chain	Involved in amino acid transport.	In GBM, SLC3A2 is highly overexpressed [49].
SPTBN1	A0A087WUZ3	Spectrin beta chain, non-erythrocytic 1	Plays a role in actin filament capping.	SPTBN1 appears to have anticancer properties and impairs cell migration. In pancreatic cancer, decreased expression of SPTBN1 is linked to a worsened prognosis [50].

#### Enrichment analysis

**Figure 4.**
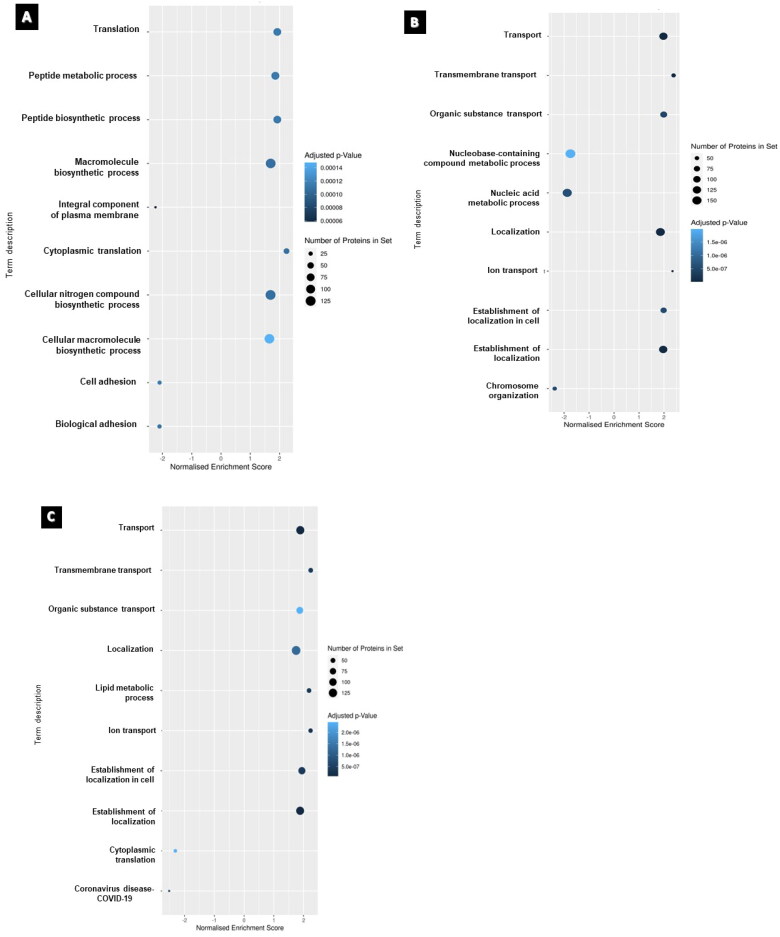
The top 10 enriched gene set enrichments for (A) cisplatin-treated, (B) paclitaxel treated and (C) combination-treated groups. Nodes are colored according to the level of significance for the enrichment and sized according to the percentage of observed proteins associated with the corresponding term that were deregulated. Note: a gene set overlapping with those sets included in the recently added COVID-19 database resulted in COVID-19 as an output and does not necessarily correspond to sample contamination.

From the obtained dataset, gene set enrichment analysis (GSEA) was performed to identify enriched pathways that could provide insights into the biological functions and mechanisms underlying the observed gene expression changes. The gene sets utilized in GSEA on proteomics data were derived from functional annotations of proteins provided by pathway databases like KEGG the Kyoto Encyclopedia of Genes and Genomes (KEGG), Reactome, and Gene Ontology (GO). These gene sets indicate functionally related groupings of proteins known to be involved in distinct biological processes or pathways. Proteomics data were first transformed to gene-level data, and each protein was linked to the gene(s) that encoded it *via* the protein sequence database Uniprot. The gene-level data were then utilized as input for GSEA analysis, which determines whether each gene set in the sample is enriched or depleted. Figure 4 illustrates the GSEA for each treatment group of the U87 cell line. The most significantly affected gene sets in the cisplatin-treated group were ‘macromolecule biosynthetic process’ and ‘cellular nitrogen compound biosynthetic process and cytoplasmic translation.’ In the paclitaxel-treated group, ‘localization’ and ‘transport’ were among the significantly perturbed, and in the combination group, ‘lipid metabolic process’ and ‘establishment of localization’ were significantly altered.

### Metabolomics analysis

An untargeted LC-MS/MS-based metabolomics analysis was conducted to investigate the activity of the metabolome post-treatment. Specific criteria were established for selection to determine valid metabolites. The first criterion was that the metabolites satisfied two key requirements: availability of MS/MS spectra and retention time (RT) or MS/MS alone. The Human Metabolome Database (HMDB-4.0) collection was used for annotation if the metabolites satisfied two key requirements. After considering additional factors, including MS/MS, RT, m/z value, and spectrum libraries, the highest annotation quality score (AQ score) was applied for the repeated metabolites, and one metabolite was ultimately chosen.

#### Differential abundance analysis

A total of 16 cisplatin-and/or paclitaxel-treated cancer cell samples, two independent biological replicates from each group (DMSO, cisplatin 9.5 μM, paclitaxel 5.3 µM, and a combination of cisplatin 9.5 μM and paclitaxel 5.3 µM) for U87 cell line were examined twice by LC-QTOF-MS, generating 131 metabolites. After excluding the exogenous metabolites (as listed in the HMDB database), 90 metabolites were included in the analyses.

#### Heatmap analysis

**Figure 5.**
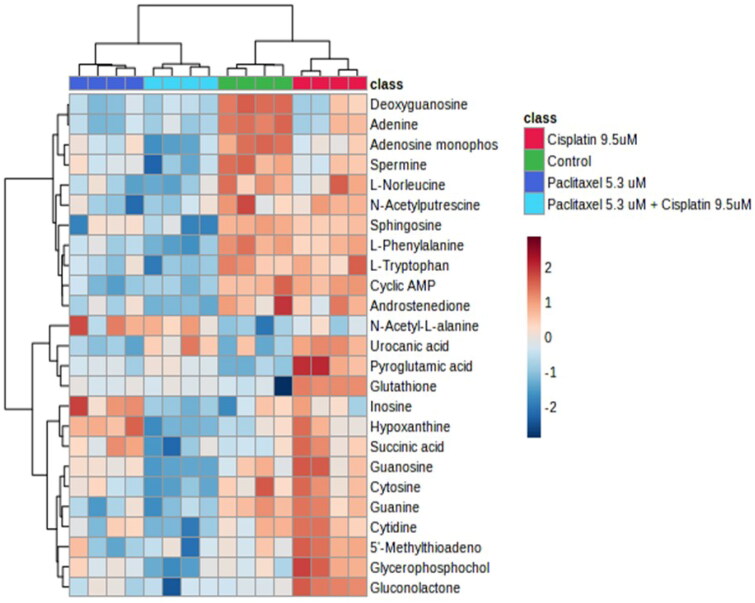
Heatmap analysis of top 25 differentially abundant metabolites. The heatmap displays the log_2_-transformed abundance levels of metabolites, along with a hierarchical clustering based on their similarity. Metabolites that were found to be significantly differentially abundant (determined by a two-sample t-test with Benjamini-Hochberg adjusted p values < 0.05 and a log2(fold-change) ≥ 1.5 for treatment vs. control) are represented in the heatmap. Highly abundant metabolites are depicted in maroon-red color, while less abundant metabolites are shown in blue color. The heatmap also includes clustering of samples, providing a visual representation of the relationships among the samples based on metabolite abundance patterns. Row headings represent gene names and colored columns represent samples indicated in the legend (Red = cisplatin, dark blue = paclitaxel, light blue = combination, green = control).

The heat map generated for the top 25 altered metabolites (Figure 5) showed complete separation between control and cisplatin, and paclitaxel and combination treatment showed no overlap (complete separation with all the groups).

#### Volcano plots

**Figure 6.**
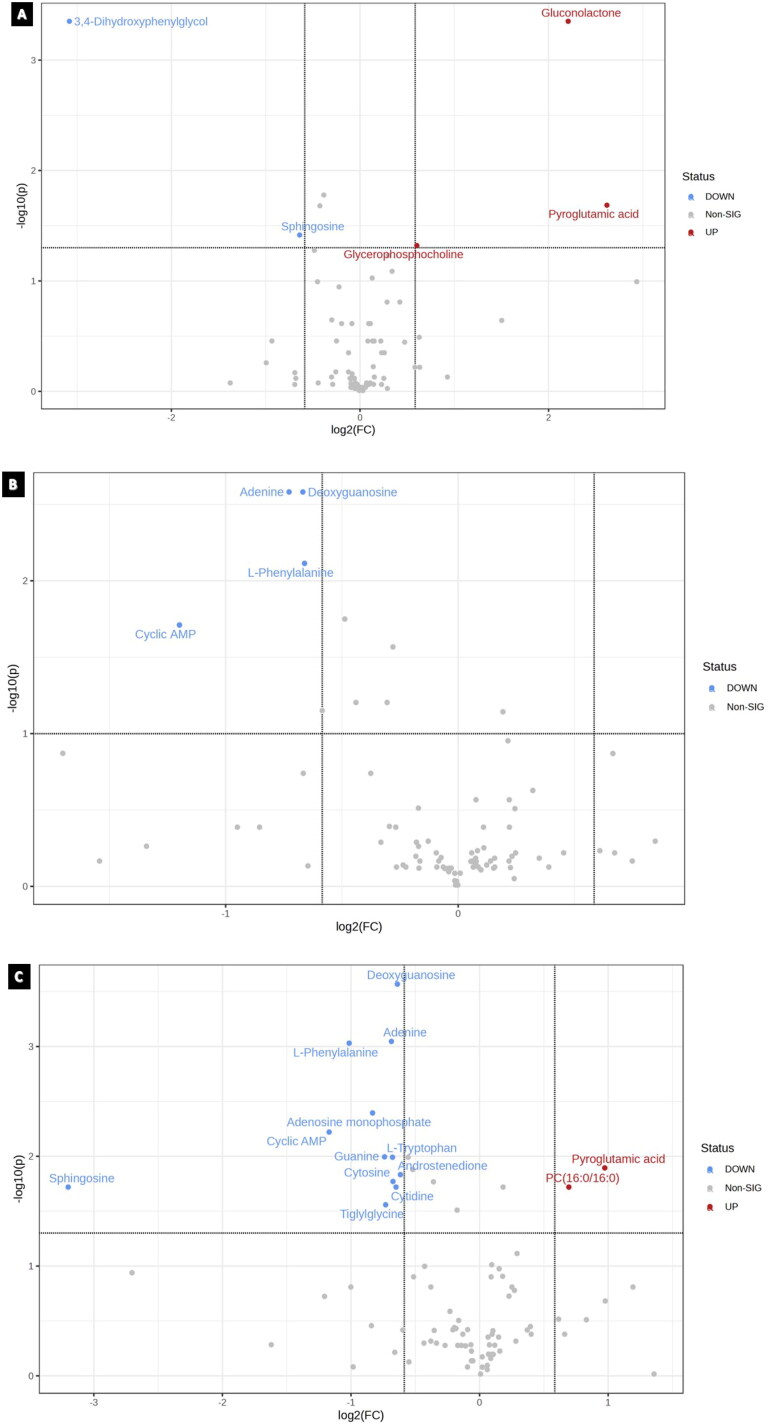
Volcano plots for the treatment groups; generated by factoring in *p*-value and foldchange of 1.5. When comparing cisplatin 9.5 µM with DMSO, a total of 5 metabolites were statistically significant., whereas a total of 4 metabolites statistically significant in the paclitaxel (5.3 μM) treated group. Nonetheless, a total of 14 metabolites were significant between cisplatin 9.5 µM with paclitaxel 5.3 µM and DMSO.

Student’s t-test was used to identify the significantly perturbed metabolites in order to analyze the alterations through volcano plots, as shown in Table 2. Volcano plots were constructed to express significantly altered metabolites (Figure 6).

**Table 2.** Results from student’s *t*-test that lists metabolites significantly altered in each treatment group with their respective *p*-values (*Exclusive metabolite in the group).

Cisplatin	Log2(FC)	*P* value	Paclitaxel	Log2(FC)	*p* value
3,4-Dihydroxyphenylglycol	−3.0816	0.00047127	Adenine	−0.72723	0.002621
Gluconolactone	2.2076	0.00047127	Deoxyguanosine	−0.66809	0.002621
Pyroglutamic acid	2.6187	0.021823	L-Phenylalanine	−0.66077	0.00769
Sphingosine	−0.63993	0.040637	Cyclic AMP	−1.1986	0.019471
Combination	Log2(FC)	p.value	Combination	Log2(FC)	p.value
Deoxyguanosine	−0.63931	0.00027	Pyroglutamic acid	0.97365	0.012756
Adenine	−0.68601	0.000899	Androstenedione	−0.61586	0.014685
L-Phenylalanine	−1.014	0.000932	Cytosine	−0.67362	0.016964
Adenosine monophosphate	−0.83223	0.004031	Sphingosine	−3.1994	0.019079
Cyclic AMP	−1.1699	0.006018	PC(16:0/16:0)	0.69424	0.019079
Guanine	−0.73899	0.010118	Cytidine	−0.64971	0.019079
L-Tryptophan	−0.67707	0.010203	Tiglylglycine	−0.73053	0.027657

#### Enrichment analysis

**Figure 7.**
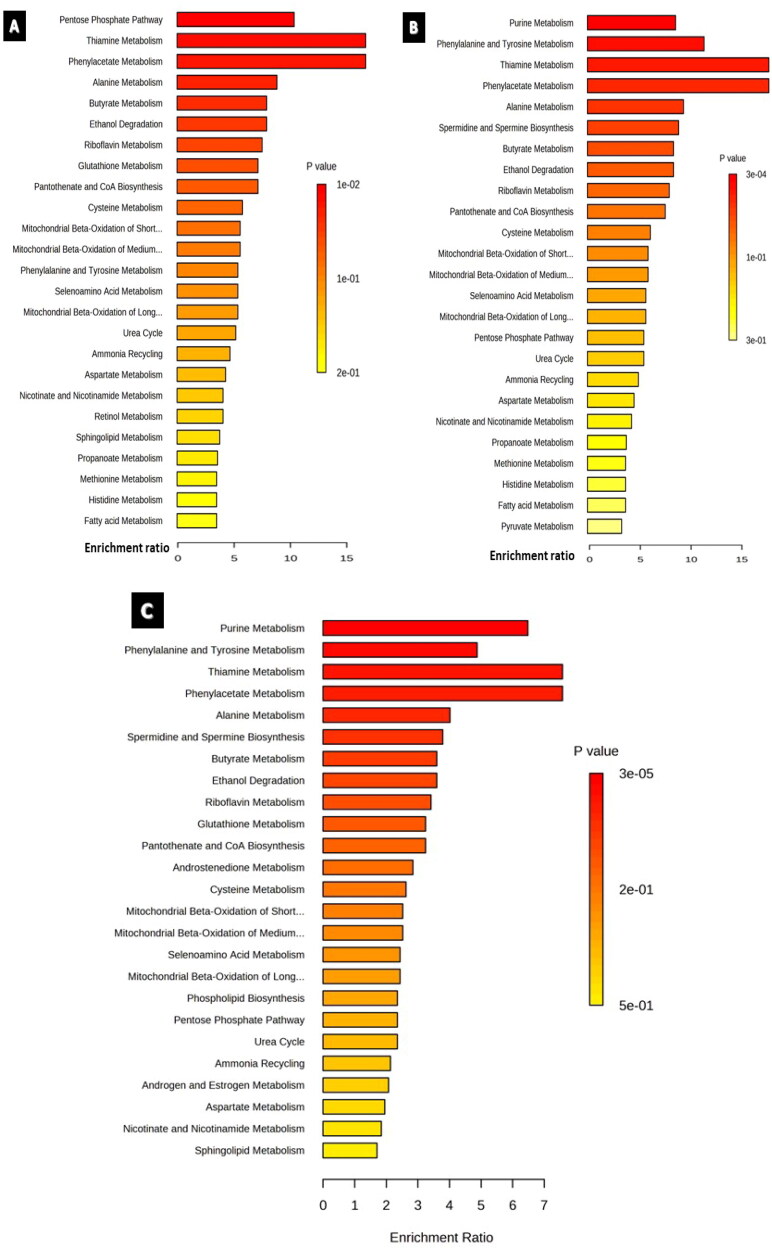
Enrichment overview of the top 25 altered metabolic pathways in U87 cell line treated with A) cisplatin 9.5 µM, B) paclitaxel 9.3 µM, C) cisplatin 9.5 µM and paclitaxel 5.3 µM. Although most enriched pathways were common between all the treated groups, androstenedione metabolism was an exclusive pathway enriched solely in the combination-treated group.

The sets of significantly altered metabolites were uploaded to MetaboAnalyst software 5.0 to test for enriched pathways defined using the Small Molecule Pathway Database (SMPBD). The results of enrichment analysis are shown in Figure 7. The pentose phosphate pathway and phenylalanine and tyrosine metabolisms were significantly enriched in the cisplatin- and paclitaxel-treated groups, respectively. In contrast, purine metabolism was significantly enriched in both the paclitaxel and combination groups.

### Multi – omics integrated analysis

**Figure 8.**
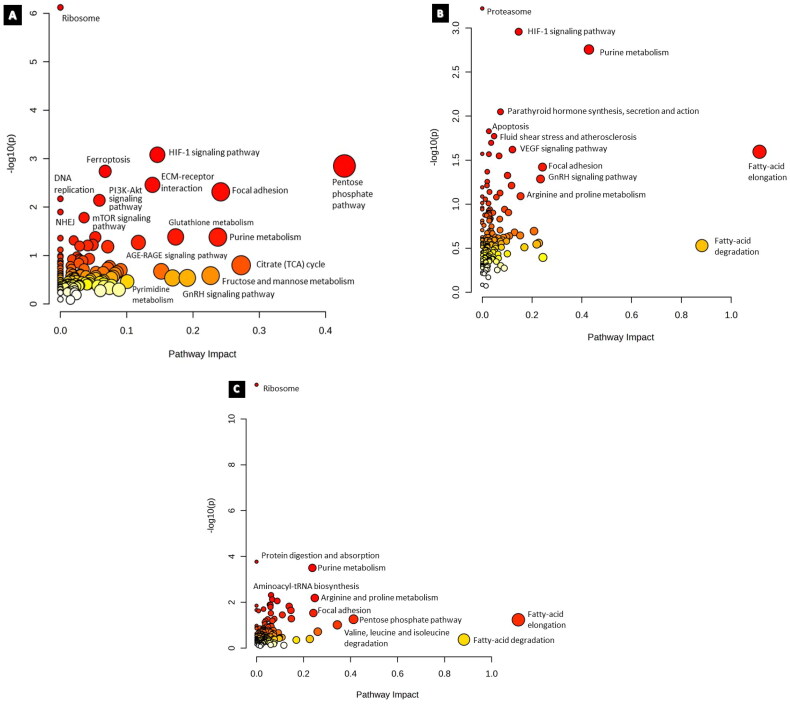
Results from joint pathway enrichment analyses. (a) cisplatin 9.5 µM (b) paclitaxel 5.3 µM (c) cisplatin 9.5 µM and paclitaxel 5.3 µM. Given the positional relevance of altered metabolites and proteins in the pathway, the pathway impact score, which is shown by the x-axis, indicates how much the entirety of the pathway is projected to be altered. The y-axis displays the pathway enrichment p values after being transformed by -log10 (higher is more significant). Nodes are sized and color coded based on the pathway effect score and *p*-value, respectively.

Results from the Joint Pathway Analysis for cisplatin (9.5 µM) treatment after the entry of 55 proteins and 7 metabolites that were significantly altered (*p* < 0.05) revealed that the impact of ribosome, Hypoxia inducible factor- 1 (HIF-1) signaling pathway, ferroptosis, and ECM-receptor interaction were altered upon treatment (Figure 8A). The input of 79 proteins and 6 metabolites from the paclitaxel (5.3 µM) group revealed significantly affected pathways of proteasome, parathyroid hormone synthesis, secretion and action, HIF-1 signaling pathway, and purine metabolism (Figure 8B). The combination group of cisplatin (9.5 µM), and paclitaxel (5.3 µM) had ribosome, protein digestion and absorption, purine metabolism, arginine, and proline metabolism (Figure 8C). Furthermore, while the HIF-1 signaling pathway and gonadotrophin-releasing hormone (GnRH) signaling pathways were enriched in both the cisplatin and paclitaxel groups, the pentose phosphate pathway and focal adhesion were common between all groups, while DNA replication and NHEJ were exclusive to the cisplatin group.

## Discussion

### The U87 cell line makes apparent attempts to mitigate the effects of cisplatin 9.5 μM treatment

#### Proteomics findings

**Figure 9.**
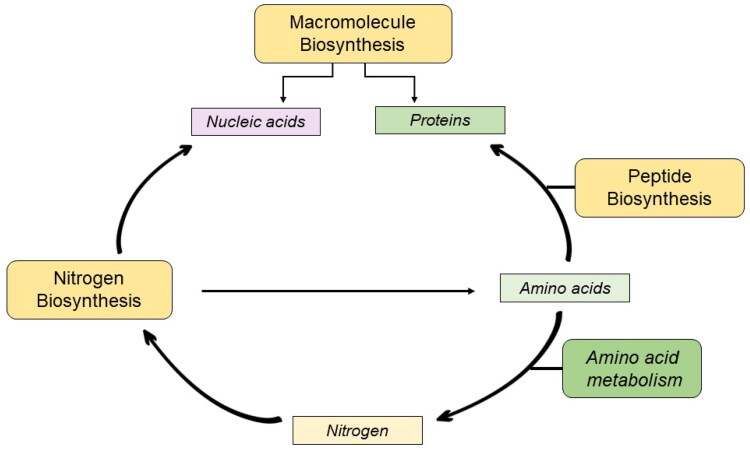
The molecular dependencies of the cellular machinery.

Proteomic analysis revealed that cisplatin treatment enriched the biosynthesis of macromolecules, peptides, and nitrogen compounds. Macromolecule biosynthesis involves the production of nucleic acids, proteins, and polysaccharides, whereas nucleic and amino acids are generated *via* the nitrogen biosynthetic process. Peptide biosynthesis involves the incorporation of these amino acids into peptide chains [51]. Enrichment of these interdependent processes (Figure 9) could lead to an overall increase in the demand for DNA repair mechanisms.

Cells must synthesize DNA repair proteins, antiapoptotic proteins, and growth-stimulating proteins to survive cisplatin treatment [52], and the functionality of ribosomal proteins is a prerequisite for this process. Consistent with this, Replication Protein A1 (RPA1) was significantly upregulated. As RPA1 recruits repair factors and regulates DNA metabolism, the enriched macromolecule biosynthesis hints at the attempt of the cellular machinery to overthrow cisplatin’s mechanism of cytotoxicity [53].

Our proteomics analysis also revealed translation as an enriched process, an intriguing finding considering cisplatin target DNA. To our knowledge, such a finding in the context of cancer has not yet been reported in the literature. However, a temporal proteomics study found that, in the model organism *Mycobacterium smegmatis*, sub-lethal rifampicin (an RNA polymerase inhibitor) doses induced phenotypic resistance through an enriched translation process [54].

Nonetheless, the enrichment of the translation process supports earlier research that proves cisplatin does not just target DNA but also affects cellular translation, which can subsequently cause polysomes to disassemble and aggregate ribosomal subunits [55]. This is because cisplatin can specifically target a number of ribosomal functional centers, indicating potential strategies by which cisplatin could hinder protein synthesis [56]. However, 40s ribosomal protein S3a (RPS3A), a structural constituent of ribosomes involved in the negative regulation of apoptotic processes and translation initiation, was significantly upregulated in this group, which could be a compensatory response to sustain translation efficiency amidst the cellular stress generated by cisplatin treatment. A potential target for future research could be ribosomal functional centers that are specifically targeted by cisplatin and their impact on protein synthesis and cellular growth. This could lead to the development of novel strategies for enhancing the efficacy of cisplatin-based chemotherapy or for identifying new therapeutic targets for cancer treatment.

Additionally, a decrease in adhesion processes has also been reported. Interestingly, adhesion serves as a distinguishing feature of cancer stem cells (CSCs) in the GBM, whereby CAMs play a crucial role in the interaction between CSCs and their tumor microenvironment, enabling cellular signaling and communication, ultimately contributing to tumor proliferation [57, 58]. Our findings on the downregulation of adhesion molecules, such as integrin 5 (ITGBM5), that affect cell-matrix adhesion suggest that cisplatin treatment may interfere with tumor cell adhesion to the extracellular matrix, which can lead to a reduction in tumor growth and metastasis. This may be a result of cisplatin-induced apoptosis, which could decrease the ability of tumor cells to attach to the surrounding tissue to grow and spread.

#### Metabolomics findings

The pentose phosphate pathway (PPP) was the most enriched finding from our enrichment analysis, which could also explain the upregulation of gluconolactone levels, an intermediate between glucose-6-phophate (G6P) and ribose-5-phopshate (R5P). Persistent DNA lesions that inhibit transcription result in glucose rerouting *via* the PPP [59]. In parallel and alternative pathways to glycolysis, PPP generates pentoses, nicotinamide adenine dinucleotide phosphate hydrogen (NADPH), and R5P for nucleotide synthesis and glutathione homeostasis, respectively [60]. The fact that inhibition of G6PD sensitizes cells to cisplatin death [61] further reinstates the contribution of PPP in maintaining cellular homeostasis due to the formation of DNA adducts.

The key enriched pathways can be summarized as amino acid metabolism pathways. Indeed, amino acid metabolism fulfils the cellular requirement for preserving redox balance and energy production, and has been identified as the primary driver of drug resistance in tumors [51, 62]. Since amino acid metabolism is a crucial step in the production of cellular energy, this finding suggests that cells are increasing their energy requirements. The upregulation of pyroglutamic acid, an amino acid involved in the metabolism of glutathione [63], may contribute to increased energy demand. These findings emphasize the importance of investigating metabolic pathways to better comprehend cellular functions and energy requirements.

#### Integrated multi-omics findings

Interestingly, HIF-1 signaling was among the enriched pathways when data from proteomic and metabolomic analyses were integrated. According to Ai et al. glycolysis is regulated by HIF-1; a downregulatory effect on HIF-1 was observed in cisplatin-sensitive ovarian cancer cells, and vice versa in cisplatin-resistant cells [64]. According to this study, the downregulation of HIF-1 shifted aerobic glycolysis in resistant cells towards mitochondrial oxidative phosphorylation, which resulted in cell death *via* excessive generation of reactive oxygen species (ROS), and hence increased sensitivity to cisplatin-resistant cells. The study proposed that the cancer metabolism pathway, including the pentose phosphate pathway regulated by HIF-1, may be a promising avenue for addressing cisplatin resistance in ovarian cancer. Although this has been the case for ovarian cancer, additional research is required to ascertain whether the same is true for GBM.

To further dismantle this conundrum, we deduced that the inherent nature of the chosen cell line itself could contribute to the enriched resistance attempts of U87 cells. This is because the genotoxic effects of alkylating chemicals are counteracted by MGMT, and MGMT promoter methylation is the main method of MGMT gene silencing. However, the U87 cell line is MGMT-deficient [65], and such results could be observed due to synthetic lethality, where loss of one DNA repair pathway increases reliance on other repair pathways [66].

### Paclitaxel 5.3 μM induces molecular alterations involved in transport

#### Proteomics findings

Transport and localization processes were enriched in response to paclitaxel treatment. As previously established, cancer cells have a higher nutritional requirement than normal cells, and given that they must double their biomass during each cell cycle, amino acid transporters play a critical role in meeting this metabolic challenge [67]. Here, the amino acid transporter SLC3A2 was significantly upregulated, suggesting increased nutritional and antioxidant requirements after paclitaxel treatment. This is because SLC3A2 not only maintains glucose uptake and glycolysis, but PPP is also associated with the cystine transporter SLC7A11, which is essential for the synthesis of glutathione.

Since paclitaxel also affects other microtubule-dependent cellular processes, including intracellular signaling, organelle transport, and motility [68], it can trigger the production of ROS [69], whereupon cells overexpress SLC3A2. Overexpression of SLC3A2 may lead to an increase in glucose uptake and metabolism, which could provide cancer cells with the energy required for their survival and proliferation. High expression of SLC3A2 protein was found to be associated with poor patient prognosis in breast cancer, but only in the estrogen receptor-positive and triple-negative subtypes, and not in the HER2 positive subtype [70]. Therefore, monitoring SLC3A2 expression levels in GBM patients undergoing paclitaxel treatment could help identify those who may be at a higher risk for drug resistance and tumor recurrence, and targeting SLC3A2 in combination with paclitaxel treatment might be a promising strategy for cancer therapy.

SLC25A22 expression was significantly upregulated in the paclitaxel group. SLC25A22 is a mitochondrial glutamate carrier. Mitochondria play a crucial role in maintaining energy homeostasis, which is essential for optimal brain functioning. Goubert et al. aimed to determine whether mitochondrial function is affected by the absence of SLC25A22 during glioma cell invasion. Silencing SLC25A22 decreased the formation of NADPH formation in response to glutamate stimulation. In addition, this inactivation led to an accumulation of glutamate within the cell, indicating that mitochondrial glutamate transport *via* SLC25A22 is essential for astrocyte glutamate homeostasis and that paclitaxel can alter mitochondrial metabolism [71].

#### Metabolomics findings

Since a lack of glutamate levels due to upregulated SLC25A22 could lead to a global reduction in ATP, we deduced that beta-oxidation of fatty acids was enriched. This is because β-oxidation of fatty acids is a metabolic pathway that involves the breakdown of fatty acids to generate ATP [72]. Similar to the cisplatin group, the paclitaxel-treated group exhibited dysregulated levels of polyamines and nucleic acids, reaffirming the dynamic response of cellular metabolism to an external stimulus.

However, spermine and spermidine biosynthetic processes were enriched and spermine levels were downregulated. Considering the proliferative and cytoprotective effects of polyamines on cultured human cancer cells, polyamines may have procarcinogenic properties [73]. Moreover, low spermidine concentrations achieved using a competitive inhibitor have been evaluated in clinical trials for various tumor types, with moderate efficacy in non-Hodgkin’s lymphoma [74, 75]. Again, paclitaxel may hinder the proliferation and survival of cancer cells by decreasing spermine levels. Spermine also plays a critical role in DNA synthesis, cell growth, and differentiation, all of which are essential for cancer cell survival and growth [76]. Our reasoning here is that paclitaxel may improve GBM treatment outcomes by inhibiting the proliferative and cytoprotective effects of polyamines.

#### Integrated multi-omics findings

By integrating metabolite and protein data, the fatty acid elongation pathway was found to be the most enriched. Fatty acids undergo elongation, wherein they are progressively extended by two carbon units after their activation as fatty acyl-CoAs. Bensaad et al. noted that triple-negative breast cancer cells rely more on lipid droplets for beta-oxidation and energy production, whereas GBM cells rely more on glycolysis for their energy needs [77]. Fatty acid elongation is involved in the production of phospholipids in cell membranes, and lipid precursors are implicated in cellular signaling [78]. We propose that paclitaxel prevents energy production through glycolysis, which GBM cells rely upon, and to counteract this, energy production becomes dependent on beta-oxidation, which supplements the process of fatty acid elongation. Additionally, fatty acid elongation proteins have been reported to be overexpressed in the mitochondria of chemotherapy-resistant breast cancer [79]. This suggests that targeting fatty acid metabolism may be a promising strategy to overcome chemotherapy resistance in GBM cells. Further studies are needed to investigate this potential therapeutic approach. Similarly, upregulation of HMOX1, regulated by HIF-1 and hypoxia [80, 81], can help mitigate oxidative stress and limit ROS-induced damage through the Fenton reaction. We speculate that these are malignant cellular responses aimed at combating paclitaxel’s attempts to cause ROS-induced damage.

### Cisplatin 9.5 μM and paclitaxel 5.3 μM make molecular attempts to reverse resistance

#### Proteomics findings

Findings from this group suggest that a potential molecular interaction between the concurrent administration of cisplatin and paclitaxel may arise to overcome the molecular hindrances associated with the use of either cisplatin or paclitaxel as monotherapy. For example, Aspartyl-tRNA Synthetase (DARS), which is responsible for the attachment of aspartate to the appropriate transfer RNA (tRNA) molecule and ensures its proper incorporation into the growing polypeptide chain, has been positively correlated with tumor purity. Findings based on gene co-expression analysis in GBM suggest that DARS is significantly upregulated upon cisplatin treatment, which has been shown to decrease cisplatin sensitivity [42, 82]. However, this expression was downregulated in the combination-treated group. This was also the case for RPS3A, which was upregulated in the former and downregulated in the latter. These observations may imply that through the intertwined nature of GBM cellular circuitry, the mechanism of action of this combination can counteract the upregulation of DARS and RPS3A and may suggest interactions that can lead to improved therapeutic outcomes.

Furthermore, the combination exhibited a greater magnitude of adducin-1 (ADD1) downregulation than paclitaxel alone. ADD1 binds to actin and has been demonstrated to have significant functions in stabilizing the cytoskeleton of the membrane and cell-cell adhesion. It binds to mitotic spindles and plays a critical role in the accurate assembly of spindles and progression of mitosis. Depletion of ADD1 by Chan et al. led to the formation of spindles that were distorted, elongated, and multipolar with an apparent abnormality in chromosome alignment [83]. Furthermore, increased expression of ADD3, another member of the adducin family, in TMZ-resistant GBM cells and in glioma stem-like cells has previously been reported [84], implying a potential positive association between adducin and the CSC phenotype. Thus, we speculate that the downregulation of ADD1 may be a key mechanism by which this combination exerts its anticancer effects in GBM and that these treatments could sensitize GBM cells to TMZ.

#### Metabolomics findings

Both the combination- and paclitaxel-treated groups had enriched purine metabolism, which could explain why nucleotide bases were altered. This could be explained by the fact that the brain lacks the enzyme machinery required to synthesize purine nucleotides from scratch; hence, it favors nucleotide synthesis *via* the salvage pathway [85]. Because these nucleotides are essential building blocks of DNA, their decrease may indicate an increase in DNA damage. Comparatively, more bases were down-regulated in the combination group. This may be linked to the inhibition of nucleotide salvage pathways by the combination effect, which leads to a decrease in intracellular levels.

**Figure 10.**
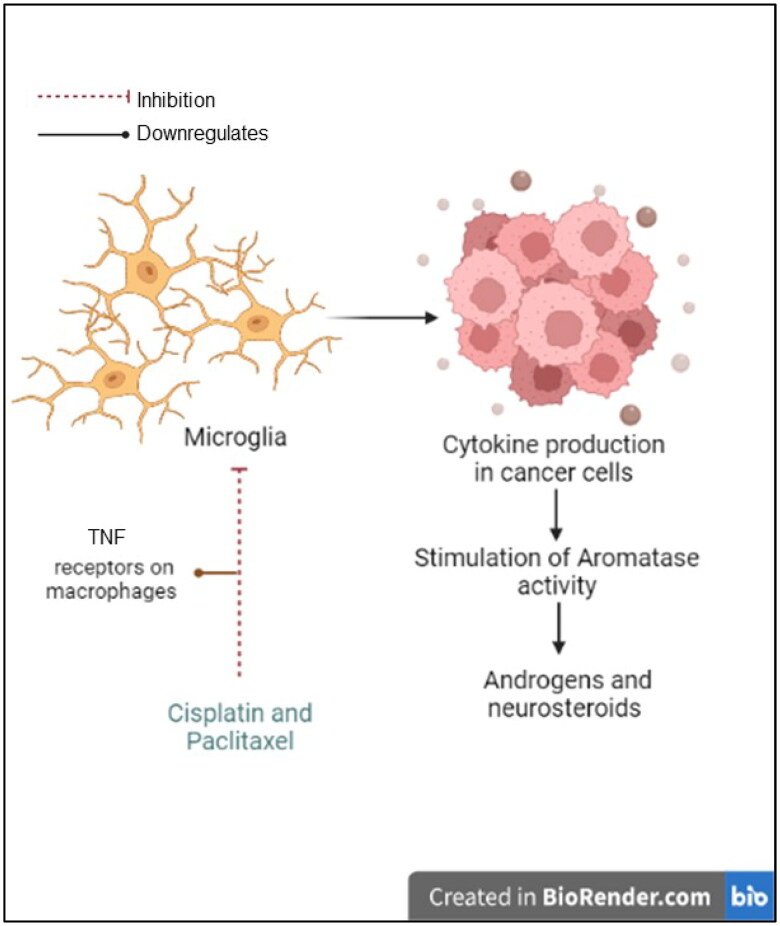
The hypothesized effect of the combined treatment on microglial activation. Diagram created with BioRender.com.

In addition to the hypothesis of a possible interaction between the drugs, the combination group exclusively exhibited enriched androstenedione metabolism owing to downregulated androstenedione levels. In addition to inhibiting microtubules, paclitaxel downregulates tumor necrosis factor (TNF) receptors on macrophages. This is significant because immune cells that infiltrate cancer cells can lead to the production of cytokines, which in turn stimulates aromatase activity [86] (Figure 10). Moreover, U87 cells retain steroidogenic enzymes in glial cells, such as 17-20α-hydroxylase, 17β-hydroxysteroid dehydrogenase and 5α-reductase [87], equipping U87 cells with the machinery required to synthesize androgens and other neurosteroids that may be involved in the formation of GBM [87]. Recently, it was reported that microglia can synthesize androstenediones [88]. Downregulation of this metabolite led us to hypothesize that this combination could inhibit glioma-associated microglial activation. This finding opens new possibilities for the development of therapeutic strategies targeting microglia in glioma, although further research is needed to investigate the potential of this approach and its effectiveness in inhibiting microglial activation.

#### Integrated multi-omics findings

The joint pathway analysis of metabolomics and proteomics data following treatment with cisplatin and paclitaxel in combination demonstrated a reduction in enriched pathways compared to the single drug treatments in U87 cells. This finding is intriguing because it suggests that the combination treatment may be more effective in reducing resistance mechanisms in cancer cells than either drug alone. Further analysis revealed that energy production pathways are the only processes prevalent in response to combination treatment, which is expected in response to any given exogenous stress in cancer cells. However, this could also indicate that the cancer cells were unable to resist the combination treatment, making it a potentially more effective strategy for reducing the growth and proliferation of cancer cells. However, it is important to note that these findings are based on *in vitro* experiments and may not necessarily reflect the complex interactions that occur *in vivo*. Additionally, our hypothesis that single-drug treatments may have activated mechanisms involved in resistance would need to be tested further through additional experiments or analyses. Nonetheless, these findings provide valuable insights into the mechanisms of action of chemotherapeutic drugs and underscore the potential of multi-omics analysis as a tool for identifying new strategies for improving cancer treatment efficacy.

## Conclusion

Our study employed a multi-omics analytical approach that combined proteomics and metabolomics to investigate the effects of cisplatin and/or paclitaxel treatment on GBM cells. By analyzing the changes in both protein expression and metabolite levels, we identified several key proteins and metabolic pathways that play important roles in the cellular response to chemotherapy. One of our key findings was that several proteins known to be involved in cisplatin resistance were upregulated in cancer cells treated with cisplatin alone but were downregulated when cisplatin was combined with paclitaxel. This suggests a potential interaction between the two drugs, as downregulation of these proteins could contribute to increased sensitivity to cisplatin. Overall, our study highlights the power of multi-omics approaches to provide insights into the molecular mechanisms underlying the response of cancer cells to chemotherapy, while creating a molecular list of alterations that can fuel future research and guide personalized healthcare approaches for improved patient outcomes. The integrated findings obtained from this study can also contribute to the identification of potential drug targets through a systems approach and pave the way for further research on this elusive malignancy. As a prospective avenue of our research, we aim to extend our findings by introducing U87 cells into mice to induce glioblastoma *in vivo*. This future study seeks to bridge the gap between our *in vitro* observations and the complex tumor microenvironment by utilizing a xenograft model. This approach will offer valuable insights into the behavior and response of U87 cells in a physiological context, allowing for a more comprehensive understanding of GBM progression and treatment outcomes.

## Data Availability

The data supporting the findings of this study are available upon request. Owing to the absence of a DOI, interested researchers may contact Mohammad Harb Semreen at msemreen@sharjah.ac.ae to request access to the data used or generated during this research. We are committed to promoting transparency and collaboration in scientific research, and will make reasonable efforts to provide the requested data in a timely manner.

## Limitations

Our study utilized two biological replicates to perform omics analysis, which would have added more power to our findings. Moreover, this study relies on data obtained through multi-omics analysis, which is susceptible to technical and analytical variability. Furthermore, the identified proteins and metabolites may have distinct roles in various cancer types and contexts, necessitating further validation to confirm the relevance of these findings. Additionally, while the IC50 value for cisplatin (9.5 μM) was sourced from the literature, it was higher than the mean response for this drug across all cancer cell lines tested, according to the Genomics of Drug Sensitivity in Cancer (GDSC) database. However, it is worth mentioning that these findings could point at the potential of 9.5 μM being a sublethal concentration.
